# Stem cell transplant for mantle cell lymphoma in Taiwan

**DOI:** 10.1038/s41598-022-09539-5

**Published:** 2022-04-05

**Authors:** Yu-Hung Wang, Ching-Yun Hsieh, Liang-Tsai Hsiao, Tung-Liang Lin, Yi-Chang Liu, Ming Yao, Tran-Der Tan, Bor-Sheng Ko

**Affiliations:** 1 Stem Cell and Leukaemia Proteomics Laboratory, University of Manchester, Manchester, UK; 2 Division of Hematology, Department of Internal Medicine, National Taiwan University Hospital, Taipei, Taiwan; 3 Division of Hematology and Oncology, Department of Medicine, China Medical University Hospital, Taichung, Taiwan; 4 Division of Hematology, Department of Medicine, Taipei Veterans General Hospital, Taipei, Taiwan; 5 Division of Hematology, Department of Internal Medicine, Chang Gung Memorial Hospital, Linkou, Taiwan; 6 Division of Hematology-Oncology, Department of Internal Medicine, Kaohsiung Medical University Hospital, Kaohsiung, Taiwan; 7 College of Medicine, Kaohsiung Medical University, Kaohsiung, Taiwan; 8 Division of Hematology and Medical Oncology, Koo Foundation Sun Yat-Sen Cancer Center, No. 125, Lih-Der Road, Pei-Tou District, Taipei, 112 Taiwan; 9 Department of Hematological Oncology, National Taiwan University Cancer Center, No. 57, Lane 155, Section 3 of Keelung Rd, Taipei, 100 Taiwan

**Keywords:** Cancer, Stem cells, Medical research, Oncology, Cancer, Haematological diseases, Prognosis, Stem-cell therapies

## Abstract

Mantle cell lymphoma (MCL) is a B-cell lymphoma featuring an aggressive course and a progressive relapsing pattern. International guidelines recommend early consolidative autologous stem cell transplant (auto-SCT) for eligible patients while reserving allogeneic SCT (allo-SCT) as therapy for refractory cases. Since data describing the implementation of transplants in the Asian population with MCL are limited, we aimed to analyze post-SCT outcomes of 99 MCL patients from the Taiwan Bone Marrow Transplant Registry database. The median age was 56 years, and 11% of the patients had blastoid variant MCL. Ninety-four patients received auto-SCT, while 13 patients received allo-SCT, eight of which received allo-SCT after failing auto-SCT. Before auto-SCT, 52% of the patients were in their first complete remission (CR1). Overall, 37 patients (39%) relapsed after auto-SCT. The median post-auto-SCT progression-free survival and overall survival (OS) were 43.6 months and not reached, respectively. Blastoid variant MCL, transplant not received in CR1, and disease progression within 12 months post-auto-SCT independently predicted inferior OS in multivariable analysis. The median post-allo-SCT OS was 74 months. Two patients (15%) died of MCL recurrence post-allo-SCT. Three patients with refractory diseases were salvaged with ibrutinib or venetoclax to allo-SCT. Treatment strategies incorporating novel agents warrant further optimization.

## Introduction

Mantle cell lymphoma (MCL) is a B-cell lymphoma that mostly presents with an advanced stage and frequent extranodal involvement, including bone marrow and gastrointestinal tract involvement^1–5^. The clinical course is typically aggressive, with a progressive shortening of response duration and disease-free survival after each relapse^6,7^. High-dose chemotherapy with autologous stem cell transplant (auto-SCT) has become the standard of care for eligible patients^8–10^. Nevertheless, the high chance of disease recurrence within 3–5 years, particularly in high-risk populations (i.e., patients with blastoid variant MCL or *TP53* mutation), often affects the post-SCT course and makes long-term survival challenging^11–17^. Allogeneic SCT (allo-SCT), in the meantime, remains an option for those who fail auto-SCT or have refractory disease^18,19^. Recently, in line with the promising responses to small molecule targeted agents, such as BTK and BCL-2 inhibitors, treatment strategies combining conventional therapy, auto-SCT or allo-SCT, and targeted therapies may be warranted^20–25^.

The current risk stratification and global treatment guidelines were primarily developed based on Western studies^26–29^. Data that describe the epidemiology, use of transplantation, and outcomes in Asian populations are relatively scarce^30–36^. Herein, we present a registry-based study and delineate the post-SCT outcome of 94 MCL patients in Taiwan. Pre-SCT parameters and therapy modalities were also analyzed for risk stratification and survival prediction.

## Results

### Patient characteristics

In total, 94 patients received auto-SCT, while 13 patients received allo-SCT (8 of which received the allo-SCT after relapsing after auto-SCT). The median age of the 94 MCL patients who received only auto-SCT was 55 years (Table 1). There were 77 male patients and 17 female patients. The Ann Arbor stage at diagnosis in most patients was stage 4 (79.8%). Approximately three-fourths of patients (74.5%) had bone marrow involvement at diagnosis. Regarding morphology subtypes, 76 patients (87.2%) had a classic type, 10 (10.6%) had blastoid variant MCL, and 2 (2.2%) had pleomorphic variant MCL. In terms of MIPI classification, patients were stratified into low (40.7%), intermediate (39.5%), and high (19.8%) risk groups. In 18 patients with available Ki-67% data from the registry, eight patients (44.4%) had a Ki-67% equal to or higher than 30%.

**Table 1:** Clinical and laboratory features of 94 mantle cell lymphoma patients who received autologous stem cell transplant.

Clinical parameter	Number* (%)
Age^†^	55 (25–73)
**Sex**	
Male	77 (81.9)
Female	17 (18.1)
Advanced stage (III/IV)	87 (92.6)
Bone marrow involvement	70 (74.5)
**Laboratory data**^**†**^	
WBC, × 10^9^ /L	10.7 (2–19)
LDH, U/L^‡^	244 (97–4082)
**MIPI**	
Low	37 (40.7)
Intermediate	36 (39.5)
High	18 (19.8)
Unavailable	3
Blastoid variant	10 (10.6)
**Ki-67**	
< 30%	10 (55.6)
≥30%	8 (44.4)
Unavailable	76

*MIPI* mantle cell lymphoma international prognostic index, *WBC* white blood cell.

*Eight patients received allogeneic stem cell transplant due to relapse/progression of disease after autologous stem cell transplant.

^†^Median (range).

^‡^LDH, normal range was 140–271 U/L.

The characteristics of the 13 patients who received allo-SCT are presented in Table 2. The median age was 49.6 years. Three of the 13 (23%) patients had blastoid variant MCL, whereas 11 of the 13 (85%) patients had bone marrow involvement of MCL at diagnosis. Overall, 7 (54%), 2 (15%), and 4 (31%) patients had low-, intermediate-, and high-risk disease according to the MIPI classification.

**Table 2:** Clinical characteristics of 13 mantle cell lymphoma patients who received allogeneic stem cell transplant.

UPN	Sex	Age	Morphology	Stage	BM involvement	MIPI	Relapse after ASCT		Salvage therapy bridging to allo-SCT	State before allo-SCT	Survival at last follow up	Overall survival*
1	Male	44	Classic	IV	Yes	Low	Yes	4 cycles of bortezomib + rituximab + bendamustine		CR	Alive in remission	88
8	Male	61	Classic	IV	Yes	Low	Yes	3 cycles of bortezomib + rituximab + bendamustine		CRu	Death due to infection	26
13	Male	52	Blastoid	IV	Yes	Low	Nil	ibrutinib		PR	Alive in remission	28
14	Male	49	Classic	III	Nil	High	Nil	1 cycle of asparaginase + paclitaxel + gemcitabine		PR	Alive in remission	140
34	Male	48	Classic	IV	Yes	Low	Yes	8 cycles of bortezomib + rituximab + bendamustine		CRu	Alive in remission	82
36	Female	66	Blastoid	IV	Yes	High	Yes	Nil		Refractory	Death due to infection	18
41	Female	52	Blastoid	IV	Yes	Low	Yes	Venetoclax		PR	Alive in remission	25
46	Male	63	Classic	IV	Yes	High	Nil	3 cycles of Ibrutinib + rituximab + bendamustine		CR	Alive in remission	11
47	Female	36	Classic	IV	Yes	intermediate	Yes	2 cycles of R-BOMES		CR	Death due to MCL	68
52	Male	52	Classic	IV	Yes	Low	Nil	Not reported		PR	Alive in remission	61
82	Male	42	Classic	III	Nil	intermediate	Yes	1 cycle of bortezomib + mitoxantrone + dexamethasone		PR	Death due to infection	132
85	Male	47	Classic	IV	Yes	Low	Nil	Not reported		PR	Death due to MCL	61
89	Male	50	Classic	IV	Yes	High	Yes	2 cycles of rituximab + bendamustine		CR	Alive in remission	24

*CR* complete remission, *Cru* complete remission, unconfirmed, *MIPI* mantle cell lymphoma international prognostic index, *PR* partial remission, *PD* progression of disease, *R-BOMES* Rituximab, carmustine (BCNU), Vincristine, Methotrexate Etoposide, Methylprednisolone, *UPN* unlinked patient number.

*Months.

Forty-five (45.5%) of the patients had available data regarding the induction treatments for MCL. The CHOP-based (cyclophosphamide, doxorubicin hydrochloride, vincristine, and prednisolone) therapy was given to 73.3% of patients^37–39^. Other induction modalities included VR-CAP (bortezomib, rituximab, cyclophosphamide, doxorubicin, and prednisone) with or without alternating with R-DHAP (rituximab, dexamethasone, cytarabine, and cisplatin)^40,41^ in 6 (13.3%) patients, RB-based therapy (bendamustine plus rituximab)^42,43^ with or without cytarabine^44,45^ in 5 (11.1%) patients, and Hyper-CVAD (fractionated cyclophosphamide, vincristine, doxorubicin, and dexamethasone) alternating with high-dose methotrexate-cytarabine^46–48^ in 1 (2.2%) patient. By and large, 8 (17.8%) patients received high dose cytarabine, while 5 (11.1%) received bendamustine in their induction chemotherapies.

### Stem cell harvest and transplantation procedures

In Taiwan, the most commonly used regimen for stem cell harvest is etoposide, methylprednisolone, cytarabine, and cisplatin (ESHAP) with or without rituximab, followed by granulocyte colony-stimulating factor (G-CSF) administration^49^. A minority of patients received high-dose cytarabine in combination with dexamethasone and cisplatin (DHAP) or other cytarabine-based regimens before harvest^50^. All except for ten patients received BCNU, etoposide, cytarabine, and melphalan (BEAM) as the conditioning chemotherapy regimen before auto-SCT^51^. Ten patients received the Benda-EAM regimen (which substitutes the BCNU in the BEAM regimen with bendamustine) as the conditioning regimen^52,53^. The mostly employed infection prophylaxis for auto-SCT is levofloxacin 750 mg or ceftibuten 400 mg daily, administered from D0 until the day absolute neutrophil counts reached 1000 cells/mm^3^. The most used conditioning regimens for allo-SCT in Taiwan are generally categorized into myeloablative and reduced-intensity regimens. The myeloablative regimens are usually backboned with busulfan IV 3.2 mg/kg/day consecutively from day − 8 to day − 5 and cyclophosphamide IV 60 mg/kg/day on day − 3 and day − 2. In contrast, the reduced-intensity regimens are based on the following scheme: fludarabine 30 mg/m^2^/day consecutively from day − 8 to day -4, busulfan IV 3.2 mg/kg/day on day − 5 and day − 4, and cyclophosphamide IV 60 mg/kg/day on day − 2. Anti-thymocyte globulin (ATG) 4–6 mg/kg can be added as a part of the conditioning regimen in HLA-mismatched allo-SCT scenarios. Usually, cyclosporin with methotrexate is used for graft-versus-host disease (GVHD) prevention in myeloablative allo-SCT, and cyclosporin with mycophenolate mofetil is used for reduced-intensity protocols.

### Stem cell transplantation

Before auto-SCT, 49 patients (52.1%) were in their first complete remission (CR1), and 16 (17%) were in their second complete remission (CR2). Twenty-nine (30.9%) patients were in partial remission (PR), of whom 16 were in PR after 1st line therapy while 13 reached PR after two or more lines of treatment. Overall, 37 patients (39.4%) had a disease recurrence after auto-SCT at a median follow-up time of 20.9 months. Among them, 23 (62.2%) patients experienced disease recurrence within 24 months after auto-SCT whereas 18 (48.6%) has patients relapsed within 12 months post-SCT. The median time to post-auto-SCT relapse was 13.1 months (range: 0.7–84).

Among the 13 patients who received allo-SCT, 8 experienced relapses after prior auto-SCT (Table 2). Five patients had primary refractory diseases and were salvaged with various regimens to receive frontline allo-SCT. Only two (15.4%) patients had recurrence of MCL after allo-SCT and eventually succumbed to the disease. However, three patients died of infections after allo-SCT. Patient unlinked patient number (UPN) 13 had disease progression before the scheduled auto-SCT, and he was salvaged with ibrutinib and achieved PR prior to subsequent allo-SCT. Patient UPN 14 had a primary disease that was refractory to multiple lines of therapy and finally achieved PR after treatment with a combination of asparaginase, paclitaxel, and gemcitabine. He then received allo-SCT and was disease-free and alive until the last follow-up. Patient UPN 36 experienced relapse one month after her auto-SCT. Allo-SCT was used as salvage due to the rapid progression of the disease. She had acute GVHD and ensuing infection and died of uncontrolled infection; imaging and bone marrow examinations before death showed no evidence of disease recurrence. Patient UPN 41 had blastoid variant MCL and relapsed eight months after auto-SCT. Her lymphoma was refractory to ibrutinib; rituximab, BCNU, vincristine, methotrexate etoposide, and methylprednisolone (R-BOMES); and rituximab, bendamustine, and cytarabine (R-BAC). She finally attained PR with venetoclax and underwent allo-SCT. Patient UPN 46 had primary refractory disease, although we confirmed that he had nonblastoid MCL. He received ibrutinib with bendamustine and rituximab and was bridged to allo-SCT. Other details are shown in Table 2.

Interestingly, a trend of growing transplantation activity with time (52% of patients were transplanted after 2016) was observed despite the increasing age at transplant (before vs. after 2016: 51.8 years vs. 56.1 years, *p* = 0.087), which partly reflects the evolution of MCL management. Although similar proportions of patients were transplanted in CR before and after 2016 (66.7% vs. 66.6%), transplantation implemented in CR1 increased by 5% after 2016 (47.9% vs. 52.9%).

### Infections

Within 180 days of auto-SCT, 46 infection episodes were documented. Seven (15.2%) patients experienced gram-negative bacilli bacteremia, and five had gram-positive cocci bacteremia. Three patients had invasive fungal infection, and one had *Pneumocystis jiroveci* pneumonia. Notably, 12 (26.1%) patients had cytomegalovirus antigenemia, while another two patients had cytomegalovirus colitis after auto-SCT. Approximately one-tenth of patients experienced a flare-up of herpes simplex virus and subsequent varicella-zoster virus infection. Other details are provided in Supplementary Table S1.

### Maintenance therapy

After proceeding with auto-SCT, 22 (23.4%) patients received maintenance therapy. Twelve (54.5%) patients received rituximab (median duration, 14.6 months, range: 4–60.8), two of whom received rituximab and alternating bortezomib with a duration of 46.8 and 36.7 months, respectively; six (27.3%) patients used bortezomib for maintenance therapy (median duration, 12.2 months, range: 4–48.6); and four used ibrutinib (median duration, 23.1 months, range: 21.8–47).

### Ibrutinib

Ibrutinib was used at a dose of 560 mg/day in twelve patients as salvage therapy: in ten patients, ibrutinib was administered because of relapse after prior auto-SCT, and in two patients, ibrutinib was given as salvaging and bridging therapy to allo-SCT. The median duration of salvaging ibrutinib for post-auto-SCT relapse was 4.6 months (range: 1.9–19.7). Of the eight evaluable patients, the overall response rate (ORR) was 62.5%. Two (25%) patients achieved PR, 3 (37.5%) patients attained CR, 2 patients (25%) maintained stable disease, and one patient (12.5%) had disease progression despite treatment. The median time to response and the response duration were 2.6 months (range: 1.9–4.4) and 6.6 months (range: 1.3–18.6), respectively. Two patients (25%) lost their response after three months and 18 months, respectively. The most frequently encountered adverse events were bleeding (37.5%), pulmonary infection (25%), including *Pneumocystis jiroveci* pneumonia (12.5%) and pulmonary nontuberculous mycobacterial infection (12.5%), and cytopenia (25%).

## Survival

**Figure 1:**
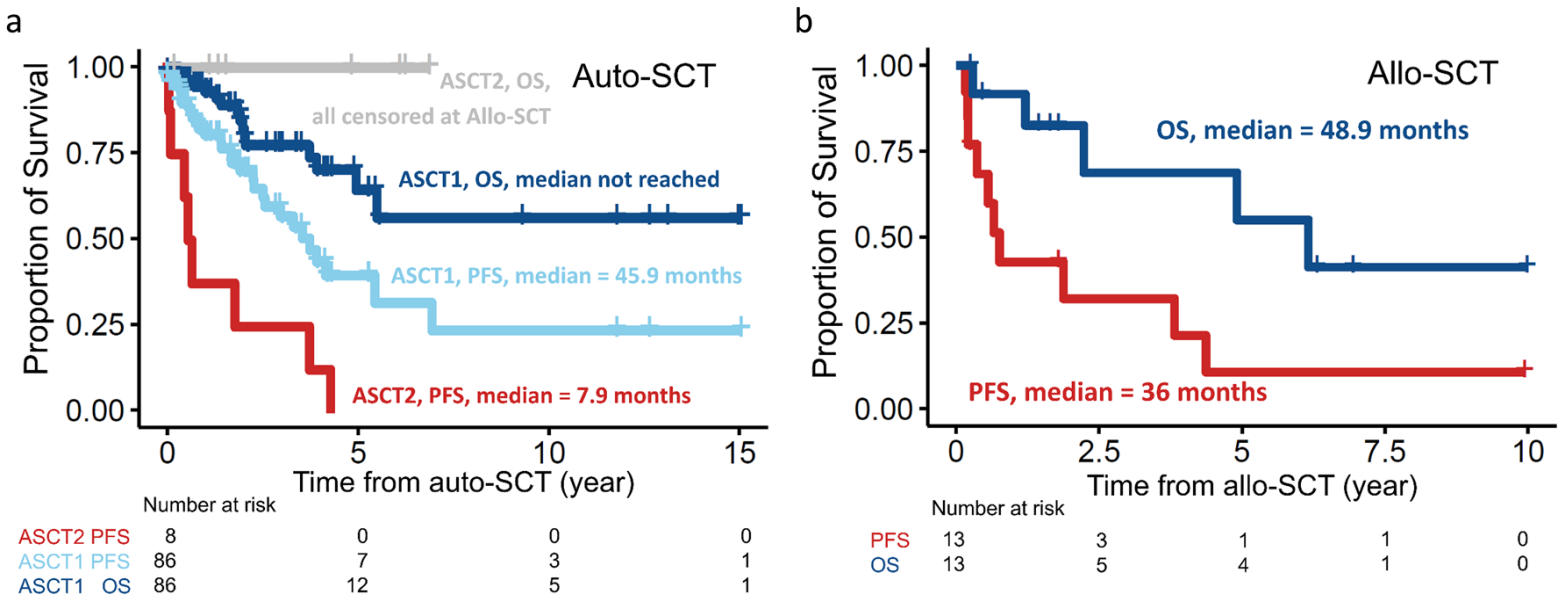
Kaplan–Meier plots of MCL patients receiving autologous (auto-) or allogeneic stem cell transplant (allo-SCT). (**a**) Progression-free survival (PFS) and overall survival (OS) of 86 patients receiving auto-SCT only (ASCT1) and 8 patients receiving auto-SCT and subsequent allo-SCT due to post-auto-SCT relapse (ASCT2). OS of ASCT2 patients were censored at the time of allo-SCT. (**b**) PFS and OS of 13 MCL patients receiving allo-SCT.

The median post-auto-SCT PFS and OS of the of 86 patients receiving auto-SCT only (ASCT1) were 45.9 months and not reached (NR), respectively (Fig. 1a). The median PFS of the 8 patients who underwent auto-SCT and subsequent allo-SCT (ASCT2) due to post-auto-SCT relapse was 7.9 months (Fig. 1a). Furthermore, the median post-allo-SCT PFS and OS of the 13 patients receiving allo-SCT were 36 months and 48.9 months, respectively (Fig. 1b).

**Figure 2:**
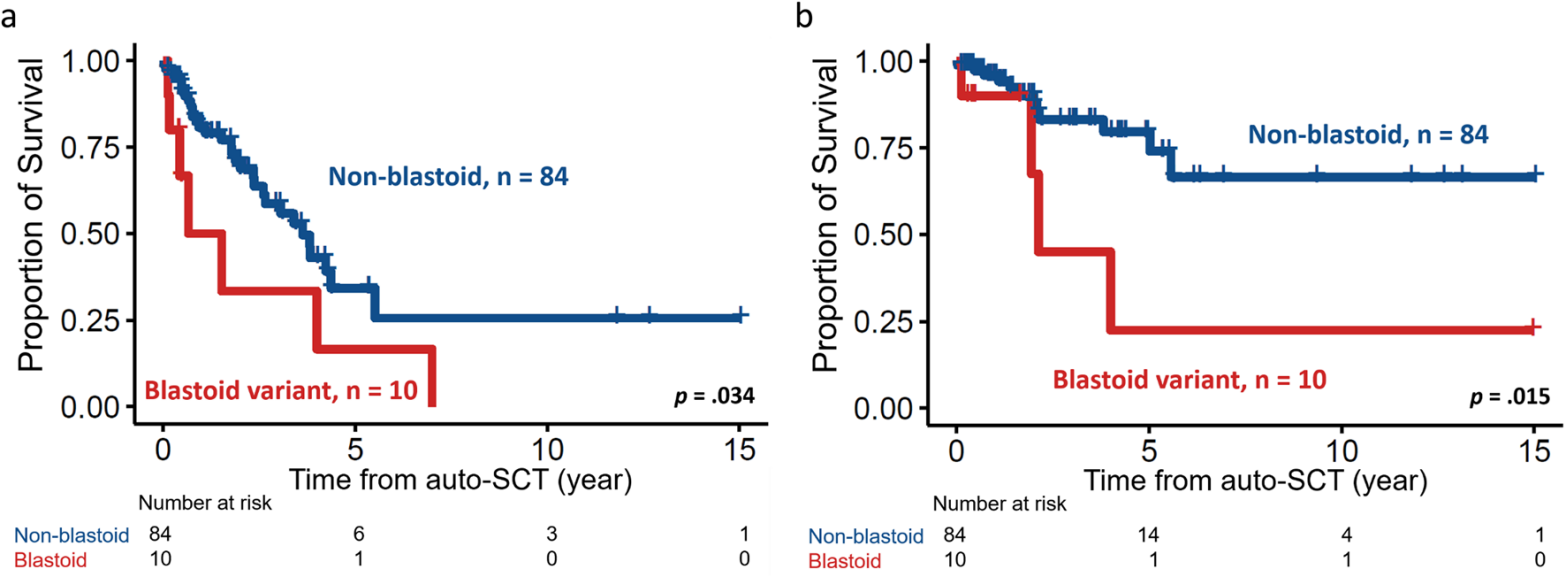
Kaplan–Meier plots stratified by morphologic variant of MCL. (**a**) PFS and (**b**) OS of 94 MCL patients with blastoid or non-blastoid variant. Patients with blastoid variant had significantly inferior PFS and OS.

**Figure 3:**
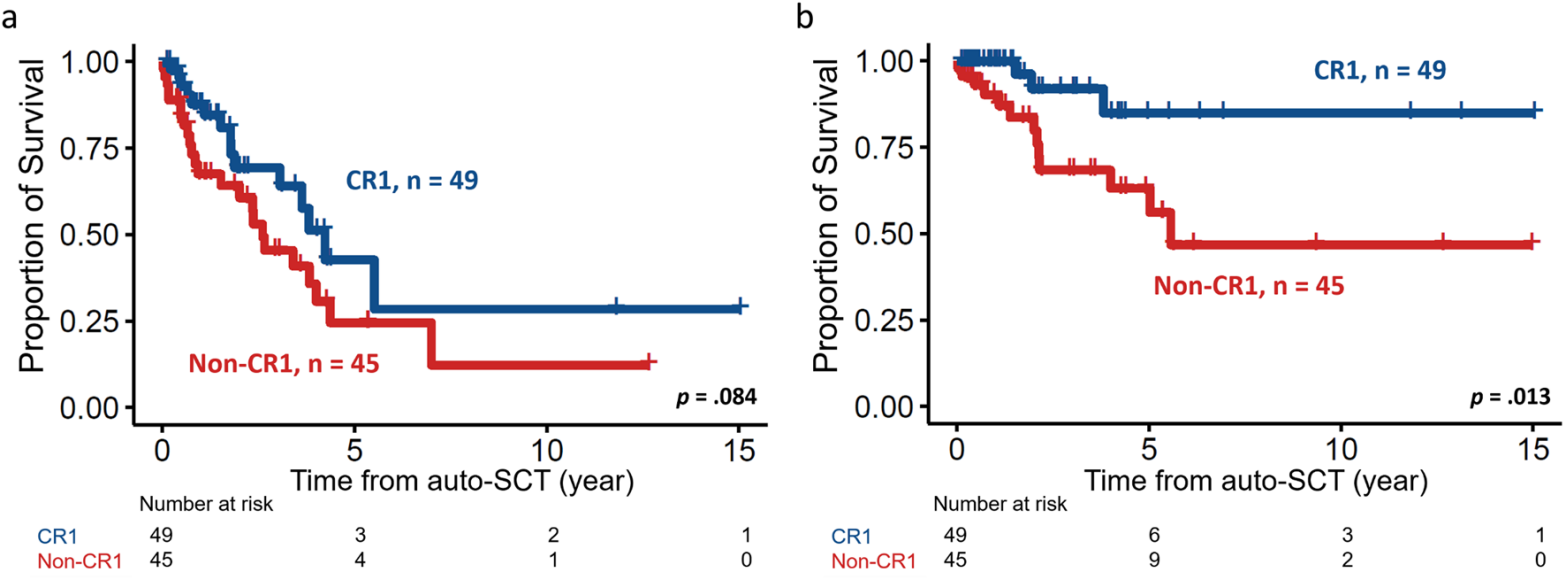
Kaplan–Meier plots stratified by disease status before autologous stem cell transplant. (**a**) PFS and (**b**) OS of patients with different disease status before receiving transplant. Patients receiving transplant at their CR1 state had better PFS and OS.

Specifically, for auto-SCT, patients with blastoid variant MCL had significantly shorter PFS and OS than those without blastoid variant MCL (median PFS, 7.9 months vs. 43.6 months, *p* < 0.001, Fig. 2a; and median OS, 25.5 months vs. NR, *p* = 0.015, Fig. 2b). Furthermore, disease status before auto-SCT also had an impact on survival, as expected. Patients who underwent auto-SCT in CR1 (n = 49, 52.1%) had more prolonged survival than those who underwent auto-SCT in CR2 or PR (PFS, 50.8 vs. 31.3 months, *p* = 0.084; and OS, NR vs. 66.8 months, *p* = 0.013, Fig. 3a,b, respectively).

**Figure 4:**
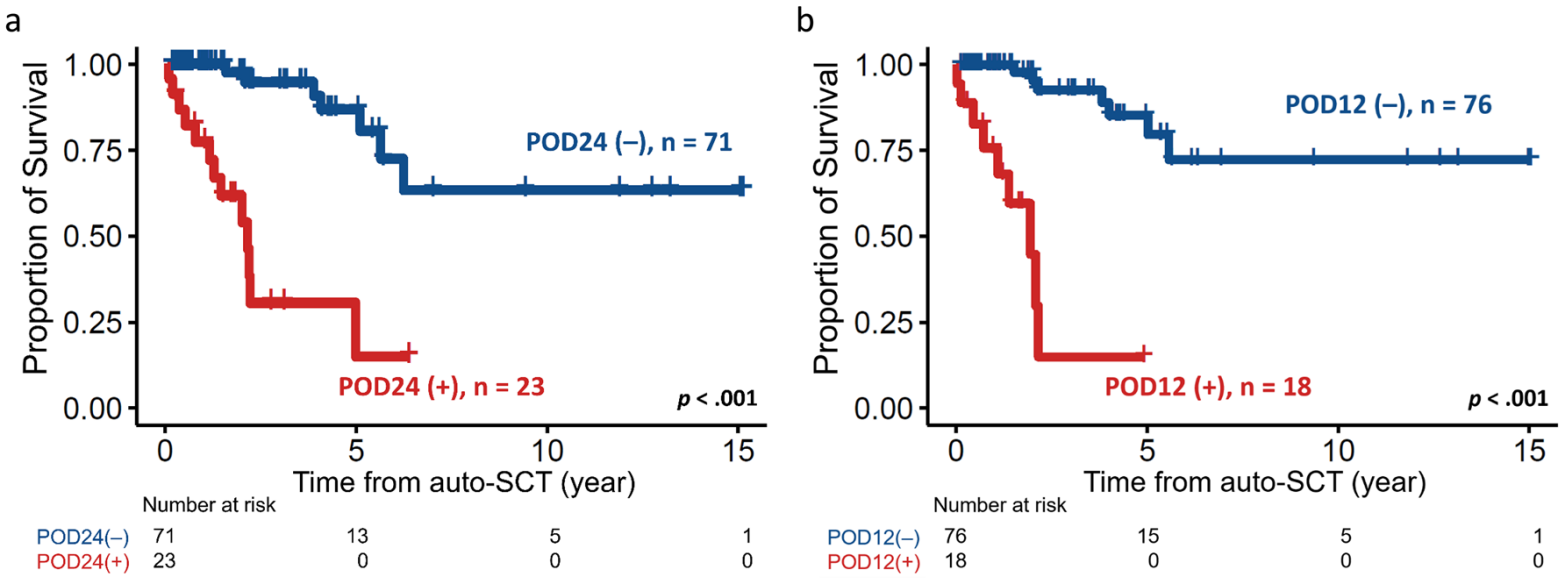
Kaplan–Meier plots stratified by progression of disease post-auto-SCT or not. (**a**) Patients who had progression of disease within 24 months (POD24) post-auto-SCT had a significantly shorter survival. (**b**) POD12 also predicted an inferior post-auto-SCT outcome.

Since time to progression (progression of disease within 24 months, POD24)^16,17^ has been established as a prognostic factor for MCL, we subsequently explored the impact of POD24 on patients’ outcomes. Conceivably, patients with POD24 had exceptionally worse survival than those without POD24 (median: 24.9 months vs. NR, *p* < 0.001, Fig. 4a). We next inquired whether progression of disease within 12 months (POD12)^14^ also affect post-SCT survival. Strikingly, patients with POD12 also had an inferior OS compared without POD12 (median: 23.3 months vs. NR, *p* < 0.001, Fig. 4b). In the multivariable analysis, blastoid variant MCL, transplant not received in CR1, and POD12 independently predicted adverse post-auto-SCT survival (Table 3).

**Table 3:** Univariate and multivariable analyses for OS of the 94 MCL patients receiving autologous stem cell transplant.

Variable	Progression-free survival				Overall survival			
	Univariate		Multivariable		Univariate		Multivariable	
	HR (95% CI)	*p-*value	HR (95% CI)	*p-*value	HR (95% CI)	*p-*value	HR (95% CI)	*p-*value
MIPI*	1.4 (0.9–2.1)	0.131	1.3 (0.8–2.0)	0.291	1.1 (0.6–2.1)	0.848	0.8 (0.3–1.9)	0.597
Bone marrow involvement	1.5 (0.7–3.2)	0.294			3.1 (0.7–13.6)	0.141		
Blastoid variant	2.4 (1.1–5.5)	0.039	1.7 (0.7–4.2)	0.260	3.7 (1.2–11.2)	0.023	3.8 (1.1–12.8)	0.033
Status before transplant^†^	0.6 (0.3–1.1)	0.088	0.6 (0.3–1.2)	0.179	0.2 (0.1–0.8)	0.023	0.3 (0.1–0.9)	0.049
Bendamustine in conditioning	0.6 (0.1–4.6)	0.641			0.1 (0–65,272)	0.666		
Maintenance therapy	0.9 (0.4–1.9)	0.712			0.2 (0.1–1.4)	0.102		
Progression within 12 months					14.3 (4.6–44.9)	< 0.001	12.7 (3.9–40.9)	< 0.001

*P* values < .05 are considered statistically significant.

Only variables with *P* value less than 0.10 in univariate analysis were incorporated into the multivariable Cox proportional hazard regression analysis.

*CI* confidence interval, *HR* hazard ratios, *MIPI* mantle cell lymphoma international prognostic index.

*MIPI: stratified into low-, intermediate-, and high-risk groups.

^†^Transplant at first complete remission versus others.

Moreover, as the recruitment of patients spanned an extended period, we further analyzed patients receiving transplantation in different eras to examine the possible chronologic effect. Patients were separated by the median calendar year of transplantation, 2016. The post-SCT outcomes of patients who received transplantation more contemporarily were better than their counterparts regarding PFS and OS (NR vs. 40.8 months, *p* = 0.039, and NR vs. 73.9 months, *p* = 0.087, respectively, Supplementary Fig. S1).

## Discussion

International guidelines recommend various induction regimens, mainly cytarabine-containing regimens, for transplant-eligible MCL patients and underscore the importance of frontline auto-SCT^28,29^. The survival benefit of early consolidation followed by auto-SCT was first reported in a prospective randomized trial of the European MCL Network, notwithstanding that there was only a PFS, but not an OS, benefit^10^. In Asia, Miura et al. analyzed the outcomes of 64 newly diagnosed Japanese patients with MCL^54^. Sixteen patients in the study received various intensive chemotherapies and ensuing auto-SCT. Before auto-SCT, nine patients were in CR1, one was in CR2, and six were refractory. The survival was rather promising, with a 5-year OS of 93%, compared to the 5-year OS of less than 50% in their counterparts. In another retrospective study, 16% of 501 Japanese patients received auto-SCT following induction chemotherapy with a rituximab-high-dose cytarabine combination^55^. This approach yielded HRs of 0.24 and 0.43 for PFS and OS, respectively, compared to R-CHOP treatment. A survival benefit of frontline auto-SCT was also observed in 97 transplant-eligible patients in a recently published Taiwanese study^32^. This multi-institutional study identified auto-SCT, gastric MCL involvement, blastoid variant MCL, and POD12 as independent prognostic factors. Patients who underwent auto-SCT in CR1 also had a better OS than those who underwent auto-SCT in CR2 or PR (median OS: NR vs. 71 months, *p* = 0.027). In accordance with previously mentioned studies, approximately half of the patients in the present study received auto-SCT in CR1 and had a superior post-SCT survival than those who received auto-SCT in CR2 or PR.

While early auto-SCT improved the long-term outcome of MCL patients, survival plateaus have not been observed. Disease recurrence frequently intercedes in the post-transplantation course, which imposes a challenge against a durable remission for MCL patients^28^. Recently, Visco et al. modelled the relationship between early POD (POD24)^16^ and risk of death in 188 relapsed or refractory MCL patients from the Fondazione Italiana Linfomi series. The early POD patients had significantly shorter OS than the late POD group (median, 12 months vs. NR, *p* < 0.0001). Early POD was also confirmed prognostically detrimental in multivariable analysis with a hazard ratio (HR) of 3.9 (95% confidence interval, 2.16–7.06) and then validated in 93 patients from the MCL Younger study. Interestingly, in 41 patients that received allo-SCT at any time during the study period, patients with early POD had comparable OS to patients with a late POD (*p* = 0.72), implying that detrimental effects of early POD might be ameliorated by allo-SCT. In a retrospective analysis of the European Society for Blood & Marrow Transplantation (EBMT) registry^56^, there was no significant difference in post-allo-SCT PFS (*p* = 0.173) and OS (*p* = 0.336) between patients with and without POD24, further demonstrating the potential of allo-SCT overcoming the poor prognosis of POD24. In the present cohort, patients with POD24 also had a reduced survival than those without, corresponding to that in Visco et al.’s study. On the other hand, although POD12 was associated with worse survival in our study, further validation in larger cohorts may be warranted.

The role of maintenance therapy with rituximab after auto-SCT has been established in the randomized, phase III LyMA trial, in which the rituximab group had superior event-free survival, PFS, and OS at 4 years post randomization^57^. A systematic review and meta-analysis also indicated that rituximab maintenance therapy improved post-auto-SCT PFS and OS in MCL patients^58^. However, post-SCT maintenance therapy in our analysis did not seem to affect post-SCT survival as much as it did in the previous studies. Admittedly, since the Taiwan National Health Insurance does not reimburse maintenance rituximab for MCL, the rate of patients receiving post-auto-SCT maintenance rituximab in this study was limited, thereby precluding a sound comparison.

While real-world data have potentially demonstrated the benefit of upfront auto-SCT in some patients, a high recurrence rate, even after consolidation treatment, has been observed in patients harboring high-risk factors, including blastoid variant MCL or *TP53* mutation^15,59,60^. Hence, an approach with intensified chemotherapy followed by frontline auto-SCT for young and fit patients might be justified, particularly in the era of novel agents and improved safety and accessibility of allo-SCT.

Although current data supporting allo-SCT as upfront therapy are lacking, earlier studies suggested at least some benefits for patients with chemorefractory MCL. Hamadani et al. investigated the outcomes of 202 patients from the Center for International Blood and Marrow Transplant Research (CIBMTR) database and found that approximately 25% of patients with refractory MCL could attain durable remission after allo-HCT^18^. In a study by Fenske et al., 519 chemotherapy-sensitive MCL patients received auto-SCT or allo-SCT with reduced-intensity conditioning at different time points during the disease course^61^. Auto-SCT and allo-SCT resulted in comparable 5-year OS rates (61% vs. 62%, *p* = 0.951). Both auto-SCT and allo-SCT in frontline settings were demonstrated to be beneficial for survival in multivariable analysis. In a recent prospective and multicenter study, 24 of 25 patients received upfront allo-SCT and were engrafted without therapy-related mortality by day 100^62^. With a median follow-up of 60.5 months, there were three (12.5%) deaths from MCL. The PFS and OS were 56% and 76% at 5 years, implying that frontline allo-SCT is feasible and should be considered for selected patients, such as those with refractory disease or a high risk of progression (i.e., POD24).

In this study, two patients had a relapse after allo-SCT, and 80% of the patients receiving upfront allo-SCT were still alive at a median follow-up of 21.5 months. Nonetheless, too few patients in this cohort received allo-SCT, hindering the results regarding the impact of allo-SCT. Therefore, larger-scale, prospective, and randomized controlled trials are warranted to support this approach.

In our cohort, the ORR of patients receiving ibrutinib for post-auto-SCT relapsed MCL was 62.5%, in agreement with that in trial settings^63–65^ as well as in real-world analysis^66^. There were no unexpected adverse events, and all events were controlled by tapering of doses. Nevertheless, the limited number of patients using BTK inhibitor could make the data much less convincing than it could be. Moreover, in the scenario where patients who remain in remission with ibrutinib and hence hesitate about SCT, it may also render bias in this transplantation registry-based analysis.

Ibrutinib has been shown efficacious in relapsed MCL after first-line treatment and auto-SCT, with ORRs spawning from 69 to 74% and CR rates ranging from 27 to 32%^56,66^. Nonetheless, its effectiveness in early-POD patients has not been conclusive. In a real-world analysis, 211 patients received ibrutinib as second-line therapy for relapsed or refractory MCL^66^. POD24 was a significant adverse predictor of PFS and OS in a univariable regression model, yet this association was lost in multivariable analysis. In Burney et al.’s study, there was no difference in the duration of response to ibrutinib between patients with and without POD24 (median: 10.4 months vs. 9.8, *p* = 0.9). After 2 years of initiating ibrutinib, the POD24 group had markedly shorter PFS and OS than patients without POD24 (*p* = 0.017 and *p* = 0.019, respectively). In the MANTLE-FIRST study^17^, curiously, ibrutinib was associated with significantly better OS in patients with POD24 compared with other second-line therapies (HR 2.41 for RB, 2.78 for R-BAC, and 2.17 for others). By contrast, in patients without POD24, such improvement in survival was not observed. As novel agents have become more broadly used in real-world practice, a treatment strategy that incorporates conventional chemotherapies, stem cell transplants, and targeted therapies needs to be optimized for this heterogeneous disease.

This study's limitations lie in its registry-based nature and, crucially, its lack of *TP53* genotyping data. The study could also be improved with more data, including data regarding the regimens and responses of each line therapy. Furthermore, this study enrolled patients over 20 years, thereby introducing various inherent confounding factors into our analysis. While early transplantation has been evoked since the early 2010s^8,14^, the lack of consensus on treatment for MCL in Taiwan makes the decision to an upfront transplantation more heterogeneous in real-world practice. Although there isn't a comparison arm for patients who did not receive transplantation in this registry-based study, a previous Taiwanese multi-institutional study indicated that 14 of 38 (37%) transplant-eligible patients received transplantation between 2006 and 2012, whereas 29 of 59 (49%) patients did between 2013 and 2019^32^. The expansion of transplantation activity for MCL, despite elder ages, reflected the remarkable integration of transplantations into the treatment strategy for MCL.

Nowadays, allo-SCT remains the curable potential in patients with relapsed/refractory MCL^61,67^. Aside from transplantation, the chimeric antigen receptor T-cells (CAR -T) may represent an alternative for long-term survival, notably after BTK inhibitors have failed^68^. In the ZUMA-2 study^68^, the ORR of 60 patients evaluated for response was 93%, with a CR rate of 67%. Remissions were durable in a majority of patients while only 15% of patients experienced grade 3 or higher but non-fatal cytokine release syndrome. In the TRANSCEND-NHL-001 Trial^69^, the ORR was 84% in 32 evaluable patients, and 59% achieved a CR. Notably, patients with a history of auto-SCT or allo-SCT were eligible in the TRANSCEND-NHL-001 study while those with prior allo-HCT was not included in the Zuma-2 study. While many trials are currently enrolling patients, the above data suggest the high efficacy of CD19-targeted CAR-T therapy for relapsed or refractory MCL, further underpinning the importance of integrating novel agents, cellular therapies, and transplantation. Consideration of disease refractoriness following relapse after transplantation or novel agents, and importantly, cost-effectiveness, is also required. Although tisagenlecleucel has been recently approved in Taiwan for the treatment of B-cell acute lymphoblastic leukemia in children and young adults^70^ and relapsed or refractory diffuse large B-cell lymphoma^71^, CAR-T therapies for MCL patients are still restricted within a clinical trial setting. However, with the impressive results from previous studies, a profound change in the treatment landscape for MCL is anticipated following the introduction of CAR-T therapy.

In summary, this study depicted the implementation of transplantation for MCL patients in an Asian population, emphasizing the survival benefit conferred by early consolidative auto-SCT compared to later auto-SCT and the prognostic impact of blastoid variant MCL and POD24. In addition, novel agents such as ibrutinib or venetoclax may play a role in bridging high-risk patients to subsequent allo-SCT, which was also demonstrated to be feasible in the frontline setting for selected patients. While the results presented are mainly confirmational, these data simultaneously convey the need for additional Asian population-based registry and clinical trials to understand this currently incurable disease and improve patient outcomes.

## Patients and methods

### Data source

This retrospective observational study reviewed and analyzed data from the Taiwan Blood and Marrow Transplantation Registry (TBMTR). The TBMTR is maintained by the Taiwan Society of Blood and Marrow Transplantation (TSBMT), which has been tasked with registering clinical information of blood and bone marrow transplant recipients in Taiwan since 2009. Currently, 17 hospitals contribute to the registry, and the collection and analysis of data from the TBMTR is approved by the institutional review board of each participating hospital. All contributing centers that were registered with the TBMTR Data Center are listed in Supplementary data S1. All experimental protocols were approved by Institutional review board or Ethical committee of Taiwan Society of Blood and Marrow Transplantation. All methods were carried out in accordance with relevant guidelines and regulations, and the Declaration of Helsinki. Informed consent was obtained from all subjects and/or their legal guardian.

### Patient selection

We recruited MCL patients consecutively from the TBMTR aged > 20 years who received one or more auto-SCT or allo-SCT for MCL between September 1999 and August 2020. A total of 99 MCL patients were identified. Data on prognosis-relevant variables, such as age, Mantle Cell Lymphoma International Prognostic Index (MIPI) classification, stage at diagnosis, morphologic type of MCL, disease status before SCT, preparation regimens, and post-SCT outcome, were extracted. Treatment response was evaluated per Response evaluation criteria in solid tumors (RECIST)^72,73^.

### Statistical analysis

We utilized the Mann–Whitney U test to compare the medians and distributions of continuous variables. Fisher’s exact test or the χ^2^ test was performed to examine the differences between discrete variables. Progression-free survival (PFS) was the duration from the date of reinfusion of autologous stem cells to the date of first documented disease progression, last follow-up, allo-SCT, or death from any cause, whichever occurred first. Overall survival (OS) was the duration from the date of reinfusion of autologous stem cells to the date of last follow-up, allo-SCT, or death from any cause, whichever occurred first. We plotted the survival curves with Kaplan–Meier analysis and calculated the statistical significance with the log-rank test. The Cox proportional hazards model was used in the univariate and multivariable regression analyses. *P* values < 0.05 were considered statistically significant. We considered biologically relevant factors (MIPI classification) and parameters with *p* < 0.1 in the univariate Cox regression analysis as covariates in the multivariable analysis. All statistical analyses and imaging were performed with IBM SPSS Statistics 23 for Windows and R software.

## Supplementary Information

Supplementary Information.

## Data Availability

The datasets generated during and/or analysed during the current study are available from the corresponding author on reasonable request.
