# Supplementary material for: Stem cell transplant for mantle cell lymphoma in Taiwan
Source: Sci Rep. 2022 Apr 5;12:5662. doi: 10.1038/s41598-022-09539-5 (PMC8983774; doi:10.1038/s41598-022-09539-5)
Supplement: Supplementary file 1 — Supplementary Information. [file 41598_2022_9539_MOESM1_ESM.pdf]

Supplement Table 1. Infections encountered by MCL patients within 1 year post autologous stem cell transplant

Category	Number (%)	Time to infection
Bacterial		
Gram positive cocci	5 (10.9)	11 (3-164)
Gram negative bacilli	7 (15.2)	4 (1-137)
Fungal		
Oral candidiasis	1 (2.2)	5
Candidemia	1 (2.2)	4
Pulmonary fungal infection*	2 (4.3)	11 (8-14)
Pneumocystis jiroveci Pneumonia	1 (2.2)	154
Viral		
Cytomegalovirus antigenemia	12 (26.1)	24 (11-126)
Cytomegalovirus colitis†	2 (4.3)	45 (35-55)
Herpes simplex virus	5 (10.9)	9 (5-32)
Varicella-zoster virus	4 (8.7)	75 (19-178)
Febrile neutropenia‡	6 (13.0)	18 (1-97)

*Probable, per the European Organization for Research and Treatment of Cancer criteria.

†With pathologic confirmation

‡Without definite culture result

Supplement Figure 1. Progression-free survival (PFS) and overall survival (OS) of patients who received transplantation before and after 2016.

(a) Patients receiving transplantation after 2016 had better PFS than their counterparts.

(b) Patients receiving transplantation after 2016 had a trend of better OS than their counterparts.

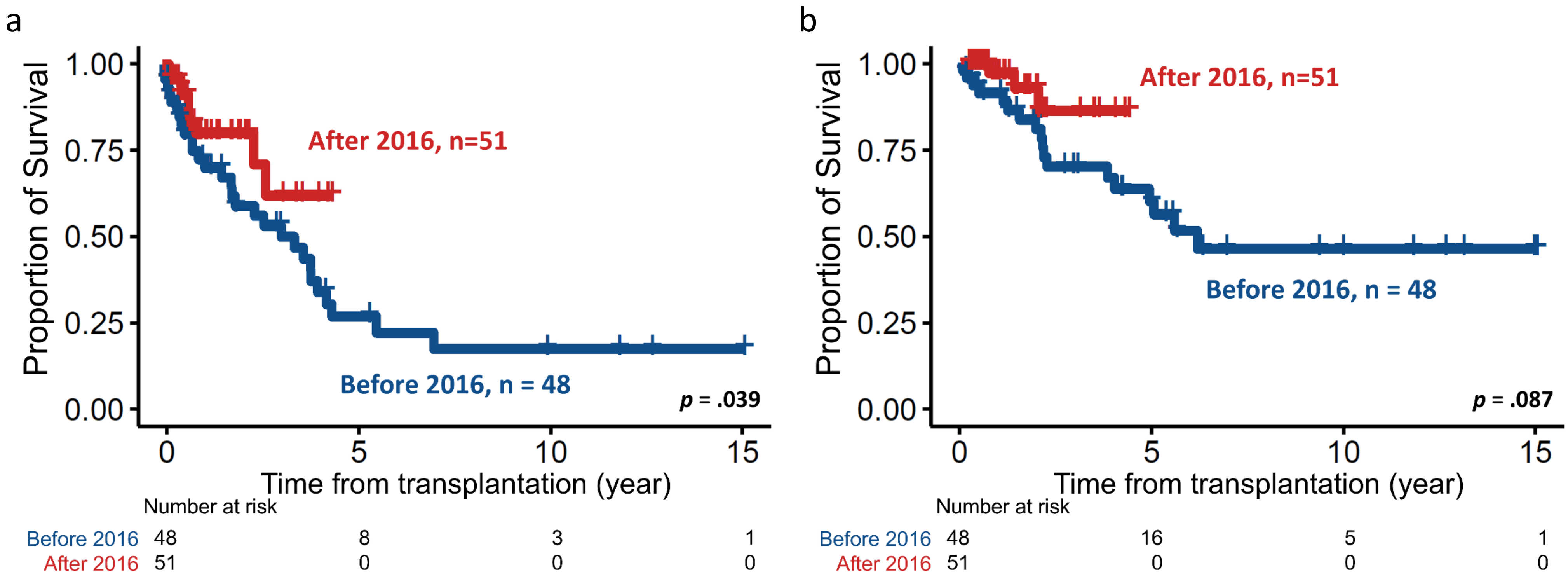

Contributing centers that were registered with the TBMTR Data Center:

Taipei Veterans General Hospital, National Taiwan University Hospital, Kaoshiung Medical University Hospital, China Medical University Hospital, Chang-Gung Memorial Hospital—Linko, Changhua Christian Hospital, Tri Service General Hospital, Koo Foundation Sun Yat-Sen Cancer Center, Hualien Tzu Chi Hospital-Buddhist Tzu Chi Medical Foundation, Chang-Gung Memorial Hospital—Chiayi, Chia-Yi Christian Hospital, National Cheng Kung University Hospital, Taichung Veterans General Hospital, Chi-Mei General Hospital, Kaoshiung Veterans General Hospital, Chang-Gung Memorial Hospital—Kaohsiung, Far Eastern Memorial Hospital
